# Bilateral international migration flow estimates updated and refined by sex

**DOI:** 10.1038/s41597-022-01271-z

**Published:** 2022-04-14

**Authors:** Guy J. Abel, Joel E. Cohen

**Affiliations:** 1 Asian Demographic Research Institute, Shanghai University, Shanghai, China; 2 Wittgenstein Centre (IIASA, VID/OEAW, WU) International Institute for Applied Systems Analysis, Laxenburg, Austria; 3 Laboratory of Populations, The Rockefeller University and Columbia University, New York, NY 10065 USA; 4 Earth Institute and Department of Statistics, Columbia University, New York, NY 10027 USA; 5 Department of Statistics, University of Chicago, Chicago, IL 60637 USA

**Keywords:** Economics, Projection and prediction, Geography, Developing world, Sociology

## Abstract

Females and males often migrate at different rates. Official data on sex-specific international migration flows are missing for most countries, prohibiting comparative measures to identify and address inequalities. Here we use six methods to estimate male and female five-year bilateral migration flows between 200 countries from 1990 to 2020. We validate the estimates from each method through correlations of several migration measures with equivalent reported statistics in countries that collect flow data. We find that the Pseudo-Bayesian demographic accounting method performs consistently better than the other estimation methods for both female and male estimated flows. The estimates from all methods indicate a decline in the share of female migration flows from 1990–1995 to 2005–2010 followed by a recovery over the decade since 2010.

## Abstract (summary)

**Table:** 

Measurement(s)	Human Migration
Technology Type(s)	R Programming Language
Factor Type(s)	origin and destination countries • five-year time periods • sex • move type (outward, return, transit)
Sample Characteristic - Organism	Homo
Sample Characteristic - Environment	global migration system
Sample Characteristic - Location	global

## Background

The sex of migrants plays an important role in many aspects of their migration experience, shaping who will migrate and where they will go, the support networks available, settlement experiences in their destination, and the strength of relationships with their origin countries. Migration data disaggregated by sex allow for a better understanding of differences in female and male migration, which can be used to identify and address inequalities. The Global Compact for Safe, Orderly and Regular Migration^1,2^ explicitly encouraged collection of sex-specific (and age-specific) migration data. Here we estimate bilateral migration flows disaggregated by sex between 1990 and 2020 to provide a global view of the past patterns and trends of female and male international migration.

Bilateral migration data measure the number of persons migrating from a set of origins to a set of destinations. As bilateral data link together two countries, they provide an empirical base to inspect migration patterns that are masked by more aggregated migration measures such as total foreign-born population sizes or net migration flows. Bilateral migration data are typically available as either measures of migrant stocks or migration flows. Bilateral migrant stock data measure the number of persons by their country of birth or citizenship and their country of residence at a specific point of time. Migration flow data measure the number of persons migrating between an origin and destination country over a given period of time.

In recent decades, sex-specific bilateral migration data have become more widely available. The United Nations Department of Economic and Social Affairs (UN DESA) regularly updates sex-specific bilateral migrant stock data. The data cover all UN member states from 1990 in five-year steps to 2020. The World Bank provides sex-specific bilateral migrant stock data for 232 countries from 1960 in ten-year steps to 2000^3^. Eurostat also regularly updates partially complete female and male bilateral stock and flow data for 42 countries in Europe and Central Asia beginning in 1990. The International Labour Organisation (ILO) provides partially complete reported bilateral migrant stock and flow statistics disaggregated by sex for working age populations for approximately 50 countries beginning in 1990. The Determinants of International Migration (DEMIG) project undertaken at the International Migration Institute collated available sex-specific data for 34 reporting countries beginning in 1946 and ending in 2011 from digitized historical national statistics and electronic resources. The Economic Commission for Latin America and the Caribbean (CELADE) provides data on international migration flows into 21 countries by sex for foreign-born populations, based on responses to related Census questions during the 1980, 1990, 2000 and 2010 rounds of censuses. Flow data measure the number of persons migrating to a destination by their country of nationality without specific details on the country of origin, rather than their country of origin (the focus of this paper).

The flow-data collections of the Eurostat, ILO, DEMIG and CELADE are based on migration statistics reported by national statistical agencies. Different countries used different definitions for counting migrants, complicating effective cross-national comparisons of migration trends and patterns. For example, there is no single common interval length for comparing persons’ countries of usual residence. Further, methods of collecting data for migration statistics vary in their accuracy. For example, data from population registers tend to be more accurate than data collected from passenger surveys at the border. Nowok, Kupiszewska & Poulain^4^ and Willekens^5^ discuss in detail the influence of measurement and definitions on the differences between reported immigration and emigration flow statistics.

The lack of comparable bilateral migration flow data has motivated recent efforts to estimate international migration flows from bilateral migrant stock data. Bilateral migrant stock data have a far greater spatial coverage and fewer problems of comparability than bilateral flow data. Several estimation methods have been proposed and separately applied to the bilateral stock data of the UN DESA and World Bank without a disaggregation by sex. Abel & Cohen^6^ used six methods and applied them to UN DESA stock data to estimate bilateral migration flows between all pairs of 200 countries in five-year intervals from 1990–1995 to 2010–2015 (and later updated on-line to include 2015–2020).

Here, we extend the research of Abel & Cohen (2019) to estimate bilateral migration flows disaggregated by sex over a thirty-year period from 1990 through 2020. In addition, we disaggregate estimates of the bilateral migration flows by the type of move (where permitted by the estimation method). The type of move may be outward migration, return migration or transit migration. Outward migrants move away from their country of birth. Return migrants move to their country of birth. Transit migrants move from and to countries, neither of which is their country of birth. Previously, Abel^7^ had estimated sex-specific migration flows based on a single method without a further disaggregation by type of move. Other applications of methods that had estimated migration flow by type of move such as those of Abel (2013), Abel and Sander (2014) or Azose and Raftery^8–10^, have not provided the detailed data sets of country-to-country estimated flows disaggregated by flow type. The new estimates presented here provide a much expanded and more up-to-date set of global female and male bilateral migration flows. We validate each set of estimates by comparisons with equivalent reported migration statistics and various commonly used migration measures.

In the remainder of this article, we first summarize six different estimation methods. We then apply each method to estimate male and female five-year bilateral migration flows over a thirty-year period. We survey our results and compare them with reported migration flow statistics. We summarise the results of our validation exercise and offer some guidance to potential users of the estimates. Finally, we discuss the limitations of our estimates.

## Methods

We summarise briefly six methods previously used to estimate global bilateral international migration flows. Details of each method can be found in the papers referenced below. In addition, Abel & Cohen (2019) compare the six methods using a standardised mathematical notation. The six methods fall into three groups. The first group includes two methods that estimate flows by differencing the bilateral stocks using different approaches for handling negative differences. The second group is a single method based on approximated migration rates. The third group includes three methods that estimate flows in a demographic accounting framework.

Stock differencing flow estimation methods require a complete table of migrant stocks born in country *i* and residing in destination *j* at time *t*, ${s}_{ij}^{t}$, and a similar bilateral migrant stock table at time *t* + 1$\left({s}_{ij}^{t+1}\right)$. The simplest method to estimate bilateral migration flows from origin country *i* to destination country over the period *t* to *t* + 1 is based on the difference between the bilateral migrant stock data; ${y}_{ij}={s}_{ij}^{t+1}-{s}_{ij}^{t}$. As the difference may be negative, researchers who have used stock differencing to approximate migration flows have either set negative differences to zero^11^ or used the negative as a proxy for the return flow from *j* to *i*^12^.

Dennett^13^ proposed to estimate global bilateral migration flows by the product of the estimated migration flow rates ${s}_{ij}^{t}/{\sum }_{hk}{s}_{hk}^{t}$ from country *i* to country *j* (using the proportions of bilateral stocks at the beginning of the period) times the global total number of migrant flows. The unknown global total number of migrant flows is approximated by the sum of the absolute net total migration flows of each country for the relevant period.

Three methods for estimating bilateral international migration flows are based on a demographic accounting framework proposed by Abel^8^. In this framework, the numbers of births and deaths in each country over the period are subtracted from the bilateral migrant stocks at the end and beginning of the period respectively. The adjusted stocks are then arranged into an array of birthplace-specific origin-destination migration flows, *y*_*ijk*_, where *i* is the origin, *j* is the destination, and *k* is the birthplace. The stocks at the beginning of the period are used as the outflow totals *y*_+*jk*_ and the stocks at the end of the period as the inflow totals *y*_*i* + *k*_. Missing values for each cell of *y*_*ijk*_ are then imputed to match these marginal constraints. The three flow estimation methods differ in how the *y*_*ijk*_ are imputed or how the adjusted stocks at the beginning and end of the period are scaled to match for each birthplace.

Abel (2013) allowed for discrepancies between the demographically adjusted migrant stock totals using an *open* demographic system whereby migration flows are estimated from or to an additional origin or destination covering countries not covered in the bilateral stock data. Alternatively, Abel & Sander (2014) formed a *closed* demographic framework by scaling the adjusted bilateral stock tables to the mid-point of their differences in their row totals.

Both Abel (2013) and Abel & Sander^9^ impute values for the array *y*_*ijk*_ in two stages. In the first stage, the diagonal cells *y*_*iik*_, which represent the number of non-migrants with birthplace *k* over a period, are set to their maximum possible values given the constraints of *y*_*i* + *k*_ and *y*_+*jk*_ formed by the adjusted stock data. In the second stage, the non-diagonal cells *y*_*ijk*_, which represent the number of migrants with birthplace *k* from origin *i* to destination *j*, are imputed using an iterative proportional fitting procedure based on a quasi-independent log-linear model to match the row, column and diagonal constraints. The final estimate of bilateral migration flows is obtained by summing the *y*_*ijk*_ array over the *k* birthplace dimension for each origin and destination.

Azose & Raftery^10^ proposed a weighted combination of two imputations for the *y*_*ijk*_ array given the constraints of *y*_*i* + *k*_ and *y*_+*jk*_ formed by the adjusted stock data from the closed demographic system. The first imputations are identical to those from the method of Abel & Sander (2014), where the diagonal elements for non-migrants are set to their maximum possible values. The second imputations use an iterative proportional fitting procedure based on an independent log-linear model to match only the row and column constraints, under the assumption that there is no difference in the distributions of migrants in the non-diagonal cells and non-migrants in the diagonal cells. The final estimate of the *y*_*ijk*_ array is obtained from a weighted combination of the two sets of imputations with a 0.87 weight in favour of the first set of imputations and 0.13 weight in favour of the second set of imputations. The weights of the two sets of imputations were estimated by Azose & Raftery (2019) using Pseudo-Bayesian comparisons with harmonised estimates of European migration flows^14^.

### Female and male estimates of bilateral international migration flows

To estimate female and male bilateral international migration flows with these six methods, we used the sex-specific migrant stocks from UN DESA^15^. The UN DESA bilateral stock data provide counts of the foreign-born populations, by place of birth, for 235 countries. The data set commences at mid-year 1990 and progresses in five-year intervals to 2020. The data are primarily based on records from censuses, nationally representative surveys and population registers provided by national statistical institutes to UN DESA. Their records are then adjusted to include refugee statistics not included in the primary data sources and to align to the mid-year time points using interpolation and extrapolations outlined in the UN DESA methodology documentation. In countries lacking data on place of birth, UN DESA use information on the country of citizenship as a proxy for the birthplace of international migrants, equating international migrants with foreign citizens in these cases. In six countries where no data on migrant totals were available, UN DESA use model countries to impute values. Details on migrant counts by sex are typically provided to UN DESA, but they note that some countries of destination do not always provide exhaustive reports of countries or areas of origin of international migrants, and hence their data might understate the number of international migrants by sex, especially for smaller countries or areas of origin. For countries or areas of destination with no data on sex characteristics of migrants, UN DESA estimates of origin-destination corridors were imputed based on a regional or country model.

The migration flow estimation methods based on rates and demographic accounting require additional data, beyond the bilateral migrant stocks. The migration rates approach relies on the sex-specific sum of countries’ net migration totals in each period. We derived these totals using the demographic accounting equation based on the sex-specific population counts at the beginning and end of the period, and the sex-specific number of deaths and births during the period. All but the last of these data are available in the 2019 World Population Prospects (WPP2019) from UN DESA^16^. We estimated the sex-specific number of births for each country during the period by multiplying the total number of births during the period by the sex ratio at birth during the period. Both of these data sets are available in UN DESA (2019)^16^. The same sex-specific population counts and the sex-specific number of births and deaths were also used to estimate female and male flows using the three demographic accounting estimation methods. As with the UN DESA migrant stock estimates, the UN DESA WPP estimates use data sources from each country plus various demographic methods to align data to common dates and impute missing values. Values are subject to potential measurement errors and WPP data on past demographic events^17,18^ and are updated every two to three years by UN DESA.

The UN DESA population estimates in WPP2019 were available for the same countries and time points as the stock estimates. The estimated net migration, birth counts, sex ratio at birth and sex-specific death counts in WPP2019 were available for 200 of the 235 countries in the migrant stock and population estimates. Each of these measures covered five-year intervals corresponding to the intervals between the migrant stock and population estimates.

We applied the six flow estimation methods (a. stock differencing drop zeros, b. stock differencing reverse negative flows, c. migration rates, d. demographic accounting of an open system with a minimisation approach, e. demographic accounting using a closed system with a minimisation approach and f. demographic accounting using a closed system and a Pseudo-Bayesian approach), given the bilateral stock and demographic estimates, to estimate migration flows over six five-year periods (1990–1995 to 2015–2020). During the last two periods, the estimates covered the 200 countries where both migrant stock and demographic estimates were available. In the first four periods, the estimates cover 197 countries. Three countries were not included in the first four periods because (1) Sudan and South Sudan, and Serbia and Montenegro are treated as single countries during these periods in the UN DESA migrant stock estimates, and (2) estimates for some methods were not feasible when including Curaçao as all UN DESA bilateral migrant stock data for Curaçao in each time point were zero. All of the 35 countries that were not included in any of the periods had populations in 2020 below 100,000. The country_list.csv file in the Figshare collection^19^ lists the countries used in each period.

**Fig. 1:**
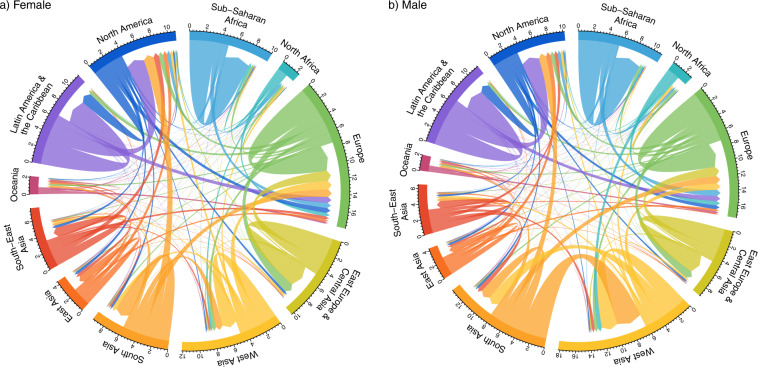
Chord diagrams of estimated (**a**) female (left) and (**b**) male (right) migration flows during 2015–2020 based on the Closed Demographic Accounting - Pseudo Bayesian methods. Direction of the flow is indicated by the arrowhead. The size of the flow is indicated by the width of the arrow at its base. Numbers on the circumferential axis, which give the size of migration flows, are in millions of individuals per five-year period. The margins of each regional sector are set to their maximum values over all five-year periods separately for each sex.

Figure 1 illustrates the female and male migration flow patterns during 2015–2020 estimated by method f, demographic accounting using a closed system and a Pseudo-Bayesian approach. The country-to-country estimated flows are aggregated to region-to-region flows, based on UN DESA regional geographies. The margins of each regional sector of the chord diagram plot are set to their maximum values over all time periods separately for each sex. Plots were produced using the circlize R package^20^. The sexes differ notably. For example, the male migration flows from South Asian countries to Western Asian countries are much larger than the female flows along the same corridors.

**Fig. 2:**
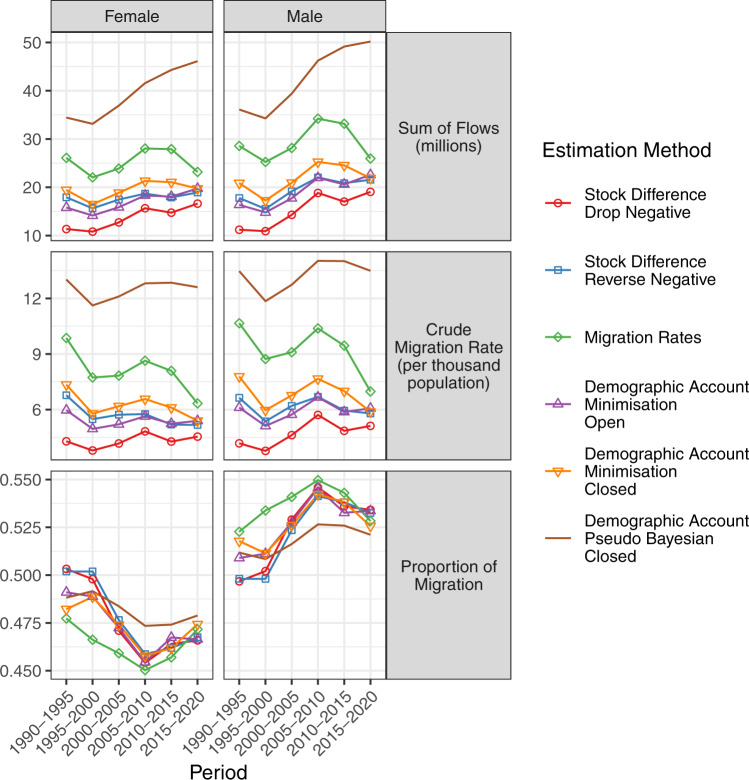
Female and male total migration flows (in millions per five-year period), corresponding crude migration rates (migrants per thousand people in the population per five-year period) and proportions of migrants of each sex for five-year periods between 1990–1995 and 2015–2020 based on six estimation methods.

The top panels of Fig. 2 show the estimated total migration flows (summed over all pairs of countries in both directions) for females and males from each estimation method over the six five-year periods. All methods estimate declining global migration flows from 1990–1995 to 1995–2000 followed by increases until 2005–2010. In the latest two periods, 2010–2015 and 2015–2020, the increase in global migration flows slows or turns negative.

The middle panels of Fig. 2 show the female and male crude migration rates for estimates from each method, obtained by dividing the total migration flows in the top panel by the total population of all countries at the beginning of the corresponding period. These crude rates follow trends similar to those of the total migration flows above.

The bottom panels of Fig. 2 show the estimated proportions (female and male) of global migration flows over the six five-year time periods. All estimated flows indicate a decline in the share of female migration flows from 1990–1995 to 2005–2010, from a peak during 1990–1995 where female migration accounts for close to half of all migration flows. In the latest two periods the share of female migration flows begins a slow recovery towards parity.

**Fig. 3:**
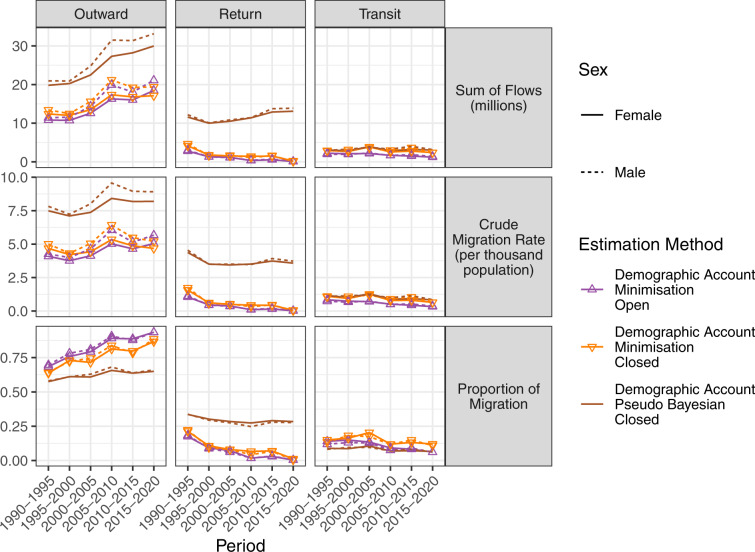
Outward, return and transit total migration flows (in millions of people per five-year period) by sex, corresponding crude migration rates (migrants per thousand people in the population per five-year period) and proportions of migrants of each sex for five-year periods between 1990–1995 and 2015–2020 based on three estimation methods.

Table 1 shows summary statistics of the flows by sex and estimation method. The proportion of estimated bilateral migration flows that are zero varies from 0.31 to 0.83, generating highly skewed distributions for flows. Figure 3 shows the total estimated migration flows for each sex by the type of flow, using the three categories introduced by Azose and Raftery^10^ to summarise global migration flow estimates from demographic accounting methods: outward, return and transit migration. Detailed estimates by the type of flow can be obtained only by the demographic accounting methods. Male and female patterns are very similar across time and migration types. The top row shows the total number of migrant flows. The middle row plots crude migration rates. Both sets of plots show that the Pseudo-Bayesian method estimates higher levels of outward and return migration than the other two demographic accounting methods.

**Table 1:** Summary statistics for bilateral migration flows over five-year periods from 1990–1995 to 2015–2020 based on six flow estimation methods.

Estimation Method	Sex	N	Minimum	Median	Mean	Maximum	Std. Dev.	Proportion Zero	Mean (non-zero)	Std. Dev. (non-zero)
Stock Difference - Drop Negative	Female	235,236	0	0	348	1,171,360	7,120	0.83	2,009	17,005
	Male	235,236	0	0	388	1,653,444	8,897	0.82	2,167	20,926
Stock Difference - Reverse Negative	Female	235,236	0	0	453	1,171,360	8,358	0.79	2,144	18,085
	Male	235,236	0	0	497	1,653,444	10,058	0.79	2,317	21,617
Migration Rates	Female	235,236	0	0	642	1,527,002	10,661	0.76	2,636	21,472
	Male	235,236	0	0	745	2,121,335	12,740	0.75	3,018	25,505
Demographic Account - Minimisation - Open	Female	235,236	0	0	433	1,296,868	8,121	0.62	1,132	13,099
	Male	235,236	0	0	485	1,591,025	9,951	0.61	1,256	15,987
Demographic Account - Minimisation - Closed	Female	235,236	0	0	497	1,093,908	8,198	0.59	1,208	12,748
	Male	235,236	0	0	556	1,406,793	9,943	0.58	1,327	15,326
Demographic Account - Pseudo Bayesian - Closed	Female	235,236	0	0	1,005	1,478,411	12,430	0.33	1,492	15,120
	Male	235,236	0	0	1,085	1,961,086	14,189	0.31	1,563	17,006

All numbers except the Proportion Zero are in units of individuals per five years. N is the number of migration corridors, where N = 235,236 = 197 origins × 197 destinations × 4 periods + 200 origins × 200 destinations × 2 periods.

The bottom row shows the relative shares of outward, inward, and transit migration flows. Outward migration flows comprise over half of the migration flows estimated by all methods. The Pseudo-Bayesian method estimates a higher proportion of return migration flows than the two other methods.

## Data Records

These bilateral migration flow estimates can be publicly accessed for free on Figshare^19^. The estimates are stored in two files. The first file contains separate rows for each sex, origin, destination and period combination (2 sexes × 197 origins × 197 destinations × 4 periods + 2 sexes × 200 origins × 200 destinations × 2 periods = 470,472) and further columns for estimates from each method: sd_drop_neg, sd_rev_neg for the estimates using stock differencing; mig_rate for estimates using migration rates; and da_min_open, da_min_closed and da_pb_closed for estimates using demographic accounting. The second file contains separate rows for each combination of sex, move type, origin, destination and period (2 sexes × 3 move types × 197 origins × 197 destinations × 4 periods + 2 sexes × 3 move types × 200 origins × 200 destinations × 2 periods = 1,411,416) with further columns for the estimates from the three demographic accounting approaches. The country names corresponding to the country three letter codes used in both files are provided in the country_list.csv file in the Figshare collection^19^.

## Technical Validation

We validate our estimated migration flows in two ways. First, we compare our estimated flows with female and male flows reported by Eurostat and the DEMIG project. Second, we compare the sum of the estimated female and male flows with flows reported by Eurostat, DEMIG and UN DESA. In the final part of this section, we discuss the discrepancies between the sum of the sex-specific flows and our previously estimated flows (Abel and Cohen 2019) from the same six methods based on the changes in the total bilateral migrant stock data with no disaggregation by sex. Details on the types of reported migration statistics for each country and period in each of collections are provided in the country_period_validation.csv file in the Figshare collection^19^.

Both comparisons for our validation use annual reported migration statistics of migration flows. However, the estimates presented above cover five-year periods due to the spacing of the bilateral migrant stock data. To compare the annual reported migration statistics to the estimated five-year migration flows, we compute equivalent five-year reported flows, in each bilateral pair and period, by multiplying the average of the annual reported migration flows during the period by five.

We do not expect the equivalent reported migration flows to be the same as the flows estimated here for two reasons. First, the annual reported migration flows use a variety of definitions that directly influence the number of migrants counted. For example, a country that defines migrants as persons who have changed their country of residence and stayed for three months will count more migrants (all else being equal) than a country that uses twelve months to define migration. Differences in definitions of migration used by countries and their impact on the level of reported international migration flows have been discussed by multiple authors^5,21–23^. Second, a five-year migration flow is likely to be less than five times an annual migration flow because of return migrants or deaths during the period. Rees^24^ discusses in detail differences between reported one- and five-year migration flow statistics.

Due to these expected discrepancies in the migration levels of the reported and estimated migration flows, we use a correlation measure for our validation rather than an accuracy measure such as mean absolute error. We calculate the Pearson’s correlation between our estimated flows and equivalent reported migration flows for six measures of migration: the count of the bilateral migration flow, the logarithm of bilateral migration flow, the proportion of bilateral migration flows into or out of a given country, the immigration and emigration rates and the net migration count. The populations in the origin and destination countries at the start of the period are used to calculate the emigration and immigration rates, respectively. These six measures for validating migration flow estimates were proposed by Abel & Cohen (2019) to cover varying potential uses for the migration flow estimates. For example, economists might use the logarithm of the migration counts in a gravity model analysis. For a population projection model, demographers might use immigration and emigration rates of migration transitions, i.e., counts of the number of persons who reside in country *i* at time *t* and country *j* at time *t* + 1.

**Fig. 4:**
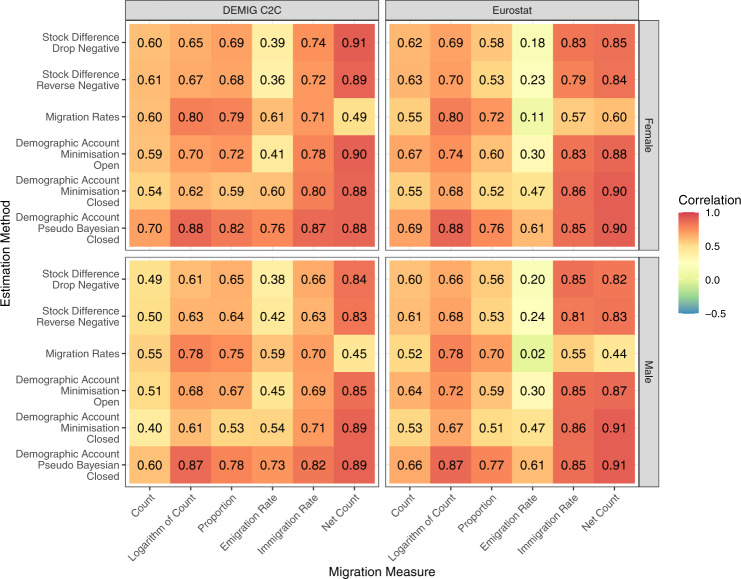
Correlations between female and male estimated migration flows during five-year periods from 1990–1995 to 2015–2020 from six estimation methods with constructed equivalent reported five-year migration flows based on the DEMIG C2C collection and Eurostat.

In our first comparison, we calculate the correlation of the estimated female and male migration flows with our construction of the equivalent reported five-year sex-specific migration flows from the DEMIG project and Eurostat. The DEMIG C2C data set^25^ provides sending and receiving sex-specific bilateral migration flow data by next or previous place of residence reported by 14 countries. We used the data to create 30,738 equivalent five-year reported sex-specific bilateral migration flows for comparison with the estimated flow. In addition, we calculated 114, 118 and 114 five-year reported sex-specific immigration rates, emigration rates and net migration counts respectively. Eurostat receiving and sending migration flow data were downloaded in March 2021 (provided in tables *migr_imm5prv* and *migr_emi3nxt* at https://ec.europa.eu/eurostat/en/). Using data from 42 European and Central Asian countries, we constructed 69,383 equivalent five-year reported sex-specific bilateral migration flows for comparison with the estimated flows. In addition, we calculated 308, 304 and 302 immigration, emigration and net migration five-year reported migration totals over the six periods. The constructed equivalent migration statistics based on the DEMIG C2C and Eurostat data were used to calculate the correlations with the estimated migration flows based on the six migration measures discussed above. The correlations are plotted in a heat map in Fig. 4.

Figure 4 shows modest differences between the correlations of female and male flows for most migration measures and estimation methods, regardless of the data source (DEMIG C2C or Eurostat). The correlations of the estimated female migration flows and the DEMIG C2C measures tend to be higher than the corresponding correlations for the male migration flows. These consistent differences suggest that the estimated methods tend to be better at capturing female migration than male migration from changes in bilateral stocks, especially for flows not involving Europe. The differences between the sex-specific correlations are more balanced from the Eurostat data. The demographic accounting methods, especially the Pseudo-Bayesian method, tend to have higher levels of correlation across all migration measures. The highest correlations, across all estimation methods, are found for the net migration counts and immigration rates. Conversely, the estimated emigration rates consistently provide the lowest correlation with the reported flows in each estimation method. The differences between the correlations from the different data sets (the left and right sides of Fig. 4) occur due to the different reported data. Eurostat covers more countries and more recent data. The DEMIG C2C data includes only two non-European countries that use previous or next place of residence when defining reported bilateral migration flows (New Zealand and South Africa).

In our second comparison, we calculate the correlation of the sum of the estimated male and female bilateral flows with the constructed equivalent reported five-year migration flows with no sex-disaggregation from five collections of reported migration data. First, the DEMIG C2C data set^25^ allowed the calculation of 17,434 five-year bilateral flows and 66, 68 and 66 immigration, emigration and net migration totals, respectively. Second, the DEMIG TOTAL data set^26^ yielded constructed equivalent five-year reported immigration, emigration and net migration totals for 63, 65 and 50 country-periods, respectively. Third, Eurostat provided 35,189 constructed five-year bilateral flows and 157, 155 and 154 immigration, emigration and net migration five-year totals, respectively. Fourth, the *International Migration Flows to and from Selected Countries: The 2015 Revision* (IMFSC2015) of UN DESA^27^ reported migration by place of residence to, from and between 39 countries, allowing us to construct 32,375 five-year bilateral flows and 172, 155 and 155 immigration, emigration and net migration totals, respectively. Fifth, the five-year net migration counts published in WPP2019^16^ were available for all 1,188 = 197 × 4 + 200 × 2 country-period combinations.

**Fig. 5:**
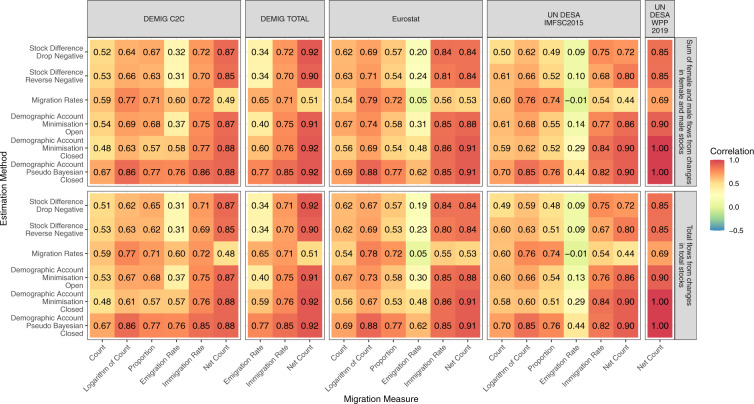
Correlations between estimated migration flows during five-year periods from 1990–1995 to 2015–2020 from six estimation methods with constructed five-year equivalent reported migration flows from five collections of migration statistics. Top panels are based on estimates from the sum of female and male migration flows described in this article. Bottom panels are based on estimates without disaggregation by sex.

We correlate the equivalent migration statistics from the five data sources with the sum of the male and female estimated migration flows from each method in the top panels of Fig. 5. The correlations of the sum of the male and female migration flows are of similar strengths to those from the sex-specific correlations for each estimation method and migration measure. Demographic accounting methods have higher correlations. The strongest correlations are in the net migration counts and immigration rates. The correlations from comparisons with the Eurostat and UN DESA IMFSC2105 are similar, as might be expected because the reported statistics come from many of the same countries. The correlations for the counts of net migration from WPP2019 are higher than the correlations from the net migration counts from the other data sets as they are based on five-year intervals, where no additional calculations were required to construct an equivalent five-year flow from annual migration statistics. There is a perfect positive correlation in all country-period combinations between the net migration counts from WPP2019 and the two methods that used the closed demographic accounting framework. These two methods use the scaling technique of Abel and Sander^9^ that constrains the estimated bilateral migration flows to sum to net migration by adjusting migrant stock totals so that difference in the total populations follow the demographic accounting equation.

For comparison, the bottom panels of Fig. 5 provide the correlations between the five data sources for the total migration flows with the estimated bilateral migration flows obtained from applying the six estimation methods to the changes in the total bilateral migrant stocks – an updated version of the estimates carried out by Abel & Cohen^6^ to the same sources of input data used in this paper. There are some minor changes in the correlations as the estimated flows from the changes in the total bilateral stock data are not constrained to match the sum of the estimated flows from changes in the female and male bilateral stock data, leading to some discrepancies between the set of estimates. Table 2 provides some summary statistics for these discrepancies between the estimated flows based on the change in the total bilateral stock data and the sum of the estimated flows of female and male flows presented in this paper.

**Table 2:** Summary statistics for discrepancies between the estimates of total migration flows from changes in the total stocks and the sum of estimates from female and male migration flows from changes in the female and male stocks.

Estimation Method	Mean	Median	Std. Dev.	IQR	IDR	IPR99	Minimum	Maximum
Stock Difference - Drop Negative	−6.4	0.0	356	0.00	0.00	40	−104,329	0
Stock Difference - Reverse Negative	−12.7	0.0	504	0.00	0.00	183	−104,329	0
Migration Rates	−13.7	0.0	342	0.00	1.35	525	−42,962	14,053
Demographic Account - Minimisation - Open	−5.6	0.0	367	0.00	0.79	587	−44,005	29,624
Demographic Account - Minimisation - Closed	−16.2	0.0	1,081	0.03	3.89	1,856	−165,180	124,121
Demographic Account - Pseudo Bayesian - Closed	−13.9	0.0	977	0.08	5.15	1,709	−149,557	108,649

IQR is Interquartile Range (the difference between the 25th and 75th percentiles). IDR is Interdecile range (the difference between the 10th and 90th percentiles). IPR99 is Interpercentile range; the difference between the 0.5 percentile and the 99.5th percentile. Units of measurement are individual migrants.

The vast majority of the discrepancies between the two sets of estimates are very close to zero. In Table 2, all the mean discrepancies, for all 6 methods, are negative. For example, for the stock difference drop negative method, the flow estimates based on changes in total migrant stocks minus the sum of male and female flow estimates is on average −6.4 persons. That is, the sums of the sex-specific estimates are on average larger than the updated aggregate estimates for all methods. The median discrepancy is zero, the interquartile ranges of the discrepancies are all less than 0.1 migrants, the inter-decile ranges of the discrepancies are less than six migrants and 99^th^ inter-percentile ranges of all the discrepancies are less than 2,000 migrants.

However, a few large differences that form long tails for the distribution of the discrepancies occur where there are simultaneously large increases in the female bilateral stock and large decreases in the male bilateral stock, or vice versa. For example, according to the UN DESA stock data, the number of Puerto Rican born migrants residing in the USA dropped by 15,028 between 2010 and 2015, leading to a migration flow estimate of zero for the period for both the stock differencing approaches. For the same period, the number of female Puerto Rican born migrants residing in the USA declined by 119,357 whereas the number of males increased by 104,329, leading to an estimated female flow of zero and male flow of 104,329. The sum of these male and female flows creates the maximum discrepancy of the stock differencing approaches shown in Table 2.

This validation provides some guidance to the relative strengths of each estimation method in relation to the migration measure of concern to the end user. There are some broad consistencies in the relative levels of correlations of each method over different data sources. The Pseudo-Bayesian demographic accounting approach tends to provide the highest levels of correlations with reported migration flow statistics. The similarities in the relative rankings of the methods over the different data sources might be due to a common set of European countries where international migration flow data are more readily available. Different levels of correlations might have been obtained if data were available from more countries and periods. In the estimated migration flows, there are 470,472 origin-destination-period combinations that could potentially form the basis of the first three (bilateral) migration measures and 1,188 origin-period or destination-period totals for the last three migration measures. In the validation exercises, equivalent reported flows from each data collection were available for less than 15 percent of the bilateral flow measures and under 28 percent of the total migration flow measures, with the exception of the total net migration from WPP2019, where all 1,188 of the corresponding reported values were available.

## Usage Notes

The estimates in this paper result from applying six different estimation methods. All methods rely on bilateral migrant stock data. The migration rates and demographic accounting methods also rely on additional measures of population changes and size. Consequently, errors and inaccuracies in stock or demographic data propagate to the migration flow estimates. The estimated migration flows from all methods are based on the number of migrant transitions over the period, i.e., a count of the number of persons who reside in country *i* at time *t* and country *j* at time *t* + 1. Migration events within the interval, such as return migration or moves to a third country are not quantified in the estimates. Counts of internal migration transitions agree closely with counts of internal migration events during a one-year period, according to multiple studies^24,28,29^. The relationship between five-year transitions and event counts are likely to be weaker because there is more time for return or moves to third countries. However, this effect is likely to be at least partially modified by the additional barriers to migration between countries compared to internal migration.

The estimated migration flows from the stock differencing methods are integers. The estimated migration flows from the migration rates and demographic accounting methods are given to two decimal places to allow users to apply statistical models that are not restricted to counts.

The best-performing methods across the different migration measures are from the two closed demographic accounting approaches. However, there is a large range in the scale of the estimated migration flows from these two methods. In the pseudo-Bayesian method, the weight calculated by Azose & Raftery^10^ is based overwhelmingly on comparisons to migration flows within Europe, where international migration is relatively easy due to freedom of movement regulations, and hence the value of the weight might push estimates of global migration flows towards the upper end of a feasible limit. In any case, we recommend that users select estimates from the method with the highest correlations based on their measure of interest and use one or more other sets of estimates from different methods to check the robustness of their results.

If users are interested solely in bilateral international migration flow estimates not disaggregated by sex, we recommend the updated on-line estimates from Abel and Cohen^6^ rather than summing over the male and female flows estimated here. The aggregation over sex provides no major improvements, as the validation exercises above show, and the estimation of sex-specific flows requires more input data, an assumption that the sex-specific input data are plausible, and an additional step in the calculation.

## Data Availability

The formatted data and R code to produce the estimates in this paper are available online in the Figshare collection^19^. The data comprise three files for the input data: the bilateral migrant stock data, the demographic changes data and the population totals. The R code comprises a single R script that a) loads the input data, b) cleans the input data to a common set of countries in each period, c) derives native-born population totals in each country required for the demographic accounting methods and d) estimates migration flows using the six different methods. The estimation functions used were developed for the migest package^30^ available on CRAN.
